# Burnout among health care professionals in medical oncology in tunisia

**DOI:** 10.1192/j.eurpsy.2021.1150

**Published:** 2021-08-13

**Authors:** A. Daldoul, W. Khechin, W. Krir, F. Ezzairi, H. Kefi, S. Zaied, S. Ben Ahmed

**Affiliations:** 1 Medical Oncology, Fattouma Bourguiba University Hospital, Monastir, Tunisia; 2 Psychiatry, Military Hospital, Tunis, Tunisia; 3 Medical Oncology, University Hospital, sousse, Tunisia

**Keywords:** burnout, oncology

## Abstract

**Introduction:**

Burnout syndrome concerns 27.8% of the general working population against 37% among doctors.

**Objectives:**

This study aimed to report the prevalence of burnout among health care professionals in medical oncology in Tunisia.

**Methods:**

This was a cross sectional study including health care professionals in medical oncology working in public hospitals in Tunisia. It was carried out from 15 January 2019 to 15 June 2019. Health profeessionals were asked to answer the Maslach –Burnout Inventory Test. Three scores allowing to locate the burn-out state of the person: the Score of emotional exhaustion (SEE), the Depersonalization score (SD) and the Score of personal achievement at work (SAP).

**Results:**

Le taux de participation était de 58,3%. La combinaison d’un SEE élevé, d’un SD élevé et d’un SAP bas définit le syndrome de Burn-out. Le SEE était élevé chez 44 travailleurs (63%), indiquant un épuisement émotionnel sévère. Un SD élevé a été trouvé chez 37 répondants (53%). La majorité des participants (59%) avaient un PAS faible. Le burnout est défini par l’association chez la même personne d’un épuisement émotionnel élevé, d’une dépersonnalisation élevée et d’un faible rendement personnel. Cela a été trouvé chez 15 des participants (21%). Selon l’étude analytique, le sexe féminin était significativement associé à un SEE élevé, un SAP bas et un épuisement global. Le travail de nuit était significativement associé à un degré élevé de dépersonnalisation et à un degré élevé d’épuisement général.

**Conclusions:**

Burnout is linked to an increasingly ergonomic load. Health care professionnel, particularly in oncology, are frequently faced to this syndrome.

